# Biodiversity and thermal ecological function: The influence of freshwater algal diversity on local thermal environments

**DOI:** 10.1002/ece3.5262

**Published:** 2019-05-22

**Authors:** Anouch Missirian, Eyal G. Frank, Jess T. Gersony, Jason C. Y. Wong, Shahid Naeem

**Affiliations:** ^1^ School of International and Public Affairs Columbia University New York New York; ^2^ Harris School of Public Policy University of Chicago Chicago Illinois; ^3^ Department of Organismic and Evolutionary Biology Harvard University Cambridge Massachusetts; ^4^ Department of Ecology, Evolution, and Environmental Biology Columbia University New York New York

**Keywords:** biodiversity, ecosystem function, global change, temperature, thermal ecology, thermography

## Abstract

The influence of temperature on diversity and ecosystem functioning is well studied; the converse however, that is, how biodiversity influences temperature, much less so. We manipulated freshwater algal species diversity in microbial microcosms to uncover how diversity influenced primary production, which is well documented in biodiversity research. We then also explored how visible‐spectrum absorbance and the local thermal environment responded to biodiversity change. Variations in the local thermal environment, that is, in the temperature of the immediate surroundings of a community, are known to matter not only for the rate of ecosystem processes, but also for persistence of species assemblages and the very relationship between biodiversity and ecosystem functioning. In our microcosm experiment, we found a significant positive association between algal species richness and primary production, a negative association between primary production and visible‐spectrum absorbance, and a positive association between visible‐spectrum absorbance and the response of the local thermal environment (i.e., change in thermal infrared emittance over a unit time). These findings support an indirect effect of algal diversity on the local thermal environment pointing to a hitherto unrecognized biodiversity effect in which diversity has a predictable influence on local thermal environments.

## 1 INTRODUCTION

The varied influences of biodiversity on ecosystem functions and properties, and the abiotic components of these systems, are well studied (Cardinale et al., 2012; Naeem, Duffy, & Zavaleta, 2012; Tilman, Isbell, & Cowles, 2014). The local thermal environment—most frequently measured as air, water, or soil temperature—which can be considered an ecosystem‐level property, however, has largely been treated as the result of extrinsic or abiotic factors such as climate, special attention being devoted to the impacts of changing temperature on biodiversity, and ecosystem properties in the face of recent, unprecedented changes in climate (IPCC, 2014). Temperature's effect on ecosystem functioning and biodiversity has been investigated: most notably, the effect of temperature change on individuals (Berry & Björkman, 1980), community diversity (Malcolm, Liu, Neilson, Hansen, & Hannah, 2006; Thakur et al., 2017), ecosystem functions (Petchey, McPhearson, Casey, & Morin, 1999), but also on many other facets of the ecosystem such as pest dynamics, niche shift, and community turnover, in terrestrial and marine systems alike, as well as the very relationship between biodiversity and ecosystem functioning, for example, (Bellard, Bertelsmeier, Leadley, Thuiller, & Courchamp, 2012; Doney et al., 2012; García, Bestion, Warfield, & Yvon‐Durocher, 2018; Logan, Régnière, & Powell, 2003; Wernberg, Smale, & Thomsen, 2012; Willis & MacDonald, 2011). The influence of biological diversity on temperature, however, is less well studied, despite temperature being an environmental parameter of fundamental ecological importance.

It is important to note that the influence of vegetation type on albedo (e.g., when boreal forest replaces grassland—see for instance; Field, Lobell, Peters, & Chiariello, 2007; Foley, Kutzbach, Coe, & Levis, 1994) is well studied. However, whether the change in plant species richness shows predictable impacts on albedo is unknown. Our focus, then, is on whether a change in the *diversity* of a given community can affect its thermal properties. Back to the albedo example for instance, whether the change in plant species richness shows predictable impacts on albedo is unknown. So is the broader influence of biodiversity on local thermal environments.

Given the roles biodiversity can play in primary productivity (Tilman, Wedin, & Knops, 1996) and other ecosystem properties [e.g., stability; (Hector, Dobson, Minns, Bazeley‐White, & Hartley Lawton, 2001; Loreau et al., 2002; Tilman, Reich, & Knops, 2006) and efficiency (Pusceddu, Gambi, Manini, & Danovaro, 2007)], biodiversity effects could translate into a change in the local thermal properties of the system, though the direction and magnitude are difficult to predict. Indeed, in terrestrial systems, for example, if more diverse communities had higher albedo or greater evapotranspiration associated with greater production, the local temperature could decrease. On the other hand, local temperature could just as well decrease in more diverse communities if increasing diversity led to increasing dominance by darker plants, hence to increased absorbance, leading to visible‐spectrum radiation being re‐emitted as thermal radiation. Germanely, diversity could have either or both of these countervailing effects in aquatic systems: increasing temperature by increasing productivity, decreasing temperature by increasing efficiency, on the top of community albedo effects.

To explore this issue, we manipulated algal biodiversity in freshwater microcosms to test for diversity effects on local thermal environments; microcosm refers to the closed system in its entirety, that is, culture vessels with their culture medium and phytoplankton community. Algal species are key members of aquatic communities that are concentrated in upper surfaces of the water column where light is abundant. They play a key role in aquatic environments as primary producers and in global biogeochemical cycles; yet, little is known about their patterns of diversity (Irigoien, Huisman, & Harris, 2004) and how they relate to primary production (Vallina et al., 2014). Because planktonic algal species contain a variety of pigments (Davies‐Colley, Pridmore, & Hewitt, 1986; Hoepffner & Sathyendranath, 1991), they absorb visible‐spectrum light (0.40–0.90 μm), some of which is used for photosynthesis, but a large portion of the remainder is re‐emitted as thermal infrared (7.5–13.0 μm), which produces sensible heat that warms the water around them. Global changes are affecting freshwater and marine communities and their diversity (Ricciardi & Rasmussen, 1999), and therefore make algal communities of additional interest from an environmental perspective. While the impacts of temperature change on algal communities, or indeed any biological community, are important as climate change increases, the role biological communities play in their changing thermal environments is unknown and could be important for understanding more clearly the two‐way interaction between temperature and ecosystems.

## 2 MATERIALS AND METHODS

We used a microcosm setup for maximal control over the variables of interest, our objective being to observe whether or not community diversity, more precisely here, species richness, has an effect on the local thermal properties of ecosystems. Thus, our focus is not on productivity (or its proxies, such as chlorophyll a or greenness): The relationship between producer diversity and production has been well studied. Rather, we focus on the effect of species richness on the radiation of thermal infrared (sensible heat), resulting from the absorption of visible light. We chose species richness as a measure of diversity to minimize the numbers of degrees of freedom and the magnitude of this novel experiment, and also to conform to the long tradition of experiments in biodiversity and ecosystem functioning (Naeem, Thompson, Lawler, Lawton, & Woodfin, 1994; Tilman et al., 1996) and thus make ours comparable to that rich body of literature. We measured sensible heat using thermography. Thermography quantifies thermal infrared radiation, in particular, that is emitted by the focal organism(s) (e.g., mammals, mollusks (Lathlean & Seuront, 2014; Seuront, Ng, & Lathlean, 2018), or, here, phytoplankton); it is distinct from greenness, which concerns reflected light (most often from chlorophyll in relatively transparent freshwater algal species).

We used algae as a model group. The algae communities consisted of 0 (control), 1, 2, 4, or 8 species drawn from a pool of eight species. These were as follows: *Ankistrodesmus falcatus*,* Chlamydomonas reinhardtii*,* Chlorella vulgaris*,* Cosmarium turpinii*,* Eudorina elegans*,* Haematococcus droebakensis*,* Selenastrum capricornutum*, and *Staurastrum gracile*. All species are freshwater algae that are commonly found in lakes and other water bodies under temperate climates, with standard nutrient and growth medium requirements (e.g., none uses silicon, and all grow at ambient temperature). We chose species that are unicellular (i.e., none were colonial, though some formed cell aggregates) and as morphologically diverse as was possible so as to maximize functional complementarity and facilitate enumeration (similar to Weis, Madrigal, & Cardinale, 2008).

### 2.1 Experimental design

Given 8 species, it is possible to form 2^8^ = 256 species combinations of any size and in particular 107 combinations of size 1, 2, 4, and 8. We explored the majority of possible combinations opting to maximize coverage of diversity rather than replication of individual combinations (see Table S1).

**Figure 1:**
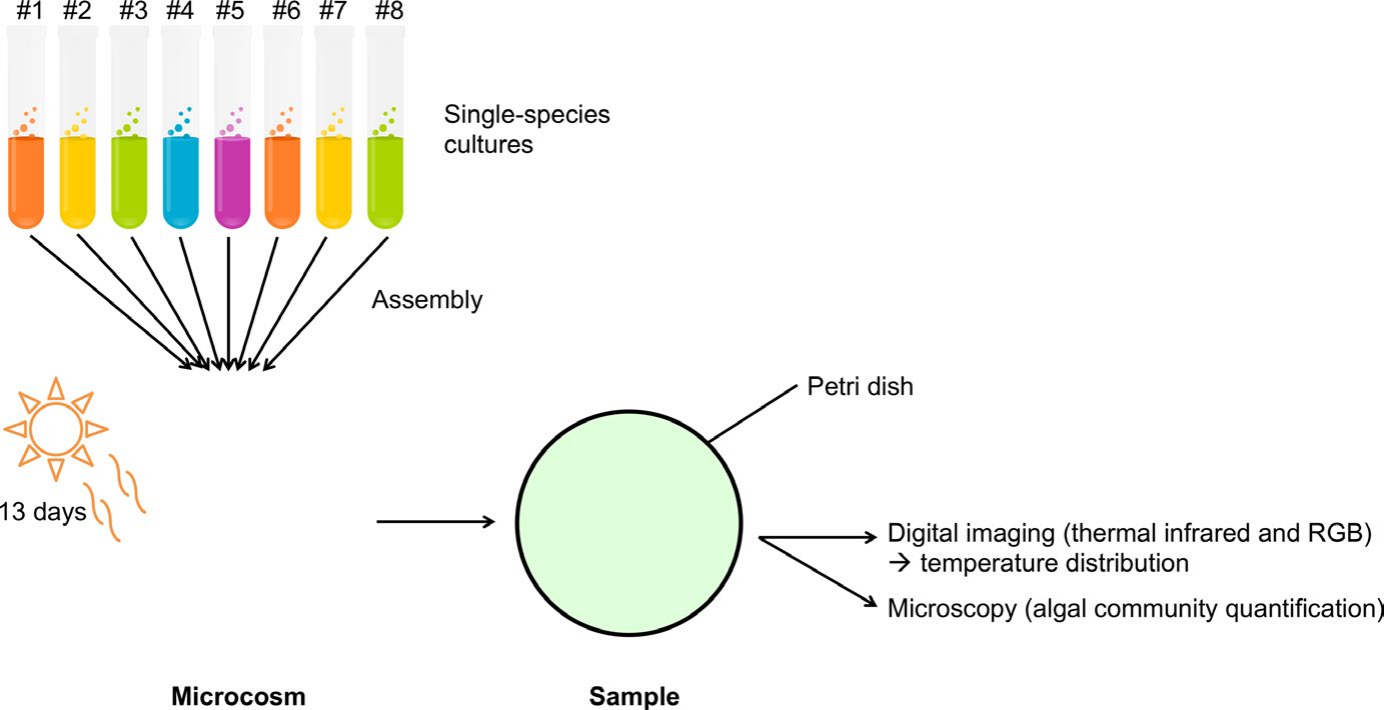
Experimental design: conceptual diagram. RGB refers to red, green, and blue sensors in visible‐spectrum camera

Specifically, we assembled 169 communities of 0, 1, 2, 4, or 8 species in transparent sterilized plastic culture flasks (15 ml, optically clear virgin polystyrene); each was labeled and filled with 1,000 cells (except the controls) and algal growth medium to total of 15 ml (Alga‐Gro^®^ Freshwater Medium, from Carolina Biological Supply Company; the algal cultures themselves were also all obtained from Carolina Biological Supply Company). Microcosms were prepared in three batches of equal size; the first one was prepared 1 week before the two others but in otherwise similar conditions, in order to facilitate sampling. The inoculation species densities of 1,000, 500, 250, and 125, for 1‐, 2‐, 4‐, and 8‐species communities, respectively, were prepared from monocultures of known densities. Finally, the microcosms were established under white, full‐spectrum lamps for 13 days (corresponding to about 13 generations) at ambient temperature (22°C); this corresponds approximately to their optimal temperature. The microcosms’ position under the lamps was randomized daily to minimize effects of possible heterogeneity in the light environment. A conceptual diagram of the experiment is presented in Figure 1.

### 2.2 Measurement

After 13 days of incubation in a nutrient‐rich environment and under constant exposure to light, the flasks were vortexed, 1 ml was used for counting (10 photographs of each slide were taken with an inverted microscope at magnification 40× for future counting), and the remaining 14 ml was used to perform thermal imagery. For thermography, the 14 ml samples were individually poured into a Petri dish, promptly covered, and exposed to fiber‐optic, low‐temperature white light (Lumina, Chiu Technical Corporation, 150 W) for 60 s (other durations were tested and yielded similar results) to allow algae to absorb light. We removed the lid and took an infrared image with a FLIR T650sc (FLIR Systems), as well as a photograph in the visible spectrum, thus measuring both temperature and visible light (RGB) reflectance of the culture (the color and opacity possibly depending on the density, health, and composition of the communities). For the second and third batches (processed together), we also took a thermal image before we heated the culture (which required removing the lid for approximately 5 s), inserting our controls at regular intervals between the samples to control for possible warming over the time it took to make measurements. This enabled us to compute Δ*T*, the temperature change before/after exposure to light (*N* = 109). The control flasks serve as a baseline for the visible and thermal imagery measurements.

### 2.3 Data processing

**Figure 2:**
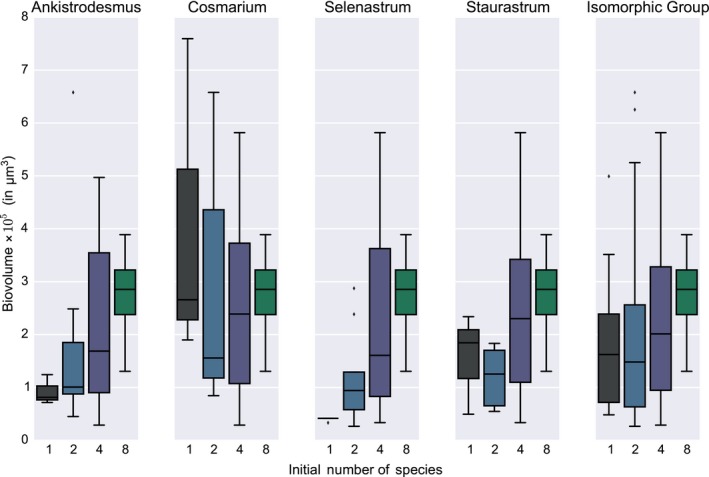
Flask biovolume per species. Each column presents the biovolumes of the flasks containing a particular species, be it as a monoculture or in an assemblage of 2, 4, and 8 species. The horizontal line corresponds to the median, the box shows the quartiles, the whiskers describe the rest of the distribution, and the points beyond the whiskers are outliers

**Figure 3:**
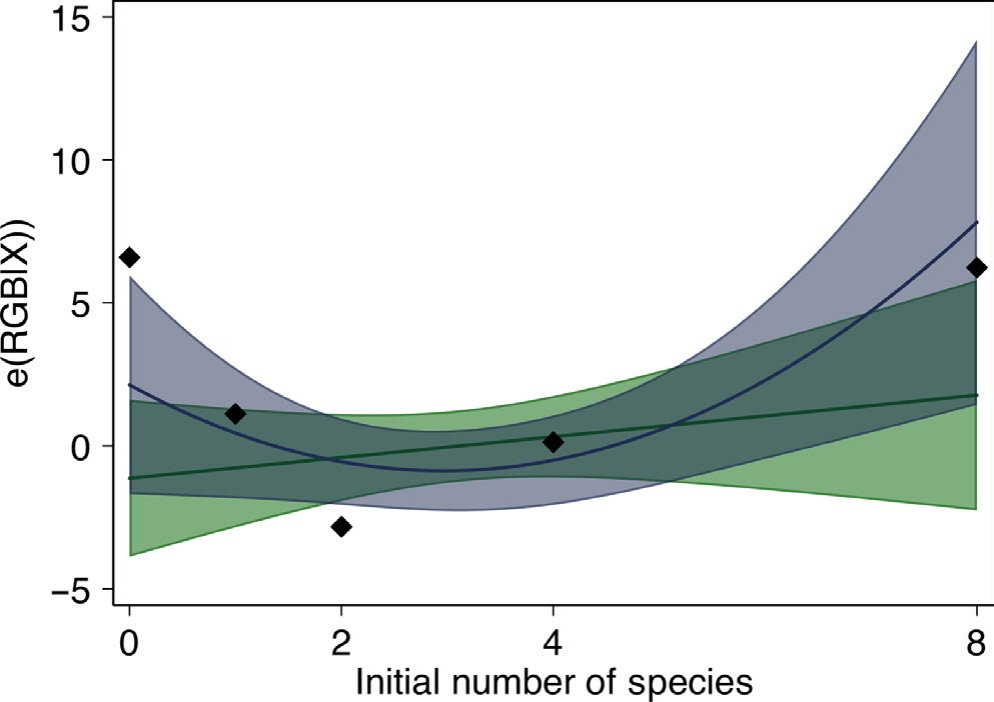
Influence of species richness on visible light reflectance. Dots represent the mean residual of RGB regressed on the dummies for the functional groups. Solid lines represent a linear fit and quadratic fit, in green and blue, respectively. Shaded areas represent 95% confidence intervals

The images obtained by optical microscopy were counted manually. Because of similar morphologies in spite of our efforts to pick dissimilar species (compare Figures S2 and S3), we were unable, in many instances, to discriminate among four species when they were in polyculture; these were *Chlamydomonas*,* Chlorella*,* Eudorina*, and *Haematococcus*. Where necessary (e.g., in the calculation of complementarity and selection effects), we therefore decided to aggregate the counts of these four species (hereafter referred to as the “isomorphic group” or IG), that is, in all measures of biovolume and cell count; because of their similar shape, size, and chloroplast density, they may share some important functional and ecological features, and obviously have a similar cell volume for purposes of biovolume estimation. From now on, “group” refers to either of the four other species or the isomorphic group (hence five groups). While removing the isomorphic group from the analysis is technically feasible, it accounts for half of the species present. Therefore, many species assemblages comprise at least one of those species (87% of our samples), and removing them would reduce the number of (noncontrol) samples on which to perform the analyses to 20 (down from 164 initially), most of which are monocultures. We therefore do not exclude them from our analyses. Nonetheless, and anticipating on the Results section, we note here that we reanalyzed the data where separating the isomorphic group was feasible, e.g. that presented in Tables 1 and 2, Figures 2 and 3, since we are using information on the initial composition or on biovolume. The complementarity and selection effects, however, are impossible to compute without lumping together the four species. This did not alter the results. For consistency, we prefer presenting the results for the four species plus isomorphic group throughout the paper.

**Table 1:** Influence of microcosm composition on biovolume. Each column corresponds to a separate regression: The independent variables are as follows: in column (1), species richness (linear); in column (2), a quadratic in species richness; in column (3), species richness (linear) and dummies (indicator functions) indicating the presence/absence of each group in the initial species mixture; and in column (4), a quadratic in species richness and the group dummies

Dependent Variable: Biovolume				
	(1)	(2)	(3)	(4)
*N*_Species_	19704.682*** (6556.075)	34765.725* (17619.573)	24697.360* (12963.029)	43228.318* (24767.124)
$N_{\mathrm{Species}}^{2}$		−2141.077 (1982.076)		−2179.164 (2244.786)
*Ankistrodesmus*			−39205.166 (26992.927)	−42324.962 (26806.441)
*Cosmarium*			49323.755 (33073.750)	46096.612 (33004.238)
*Selenastrum*			−38465.483 (27172.388)	−41133.504 (27390.329)
*Staurastrum*			−21112.737 (27379.180)	−23953.073 (27880.794)
Isomorphic group			12150.509 (44893.394)	−3248.527 (50141.158)
Constant	139032.475*** (25403.689)	118424.819*** (35827.400)	131024.894*** (36880.235)	117830.291*** (38599.427)
*R* ^2^	0.046	0.050	0.107	0.111

*N* = 162. Robust standard errors are indicated in parentheses.

* *p* < 0.1

** *p* < 0.05

*** *p* < 0.01.

**Table 2:** Regression: Species presence and biovolume effect on standardized RGB. Each column corresponds to a separate regression on standardized RGB: The independent variables are as follows: in column (1), standardized biovolume, and in column (2), standardized biovolume and group dummies (signalling the initial presence/absence of each group in the microcosm)

Dependent Variable: Standardized RGB		
	(1)	(2)
Standardized biovolume	−0.107 (0.077)	−0.140* (0.079)
*Ankistrodesmus*		−0.253 (0.167)
*Cosmarium*		0.292* (0.160)
*Selenastrum*		−0.392** (0.155)
*Staurastrum*		0.084 (0.165)
Isomorphic group		−0.292 (0.246)
Constant	0.005 (0.079)	0.353 (0.250)
*R* ^2^	0.011	0.099
*N*	162	162

Robust standard errors in parentheses.

* *p* < 0.1

** *p* < 0.05

*** *p* < 0.01.

We estimated biovolume for each group based on the optical microscope images (available in the Table S2), as data available from different sources on our species’ unitary biovolume (the volume of a single cell) seemed not to converge.

Selection and complementarity effects were measured following (Loreau & Hector, 2001). The selection effect refers to the fact that, given a set of species, a random draw from that pool may select a species with a level of function above average; and thus, by increasing diversity (i.e., here, the number of species), one increases the likelihood of picking those high‐function species. The complementarity effect, on the other hand, refers to the fact that species may occupy different ecological niches, thus improving resource use efficiency, and may in addition interact in a synergistic (or an antagonistic) way. Overall, these interactions yield a level of function different from what might have been expected by extrapolating function from the monocultures.

The temperature and RGB (visible spectrum) profiles were extracted from each infrared image using FLIR ExaminIR and ImageJ (Rasband, 19972014) software, and operations on data were conducted in Python with the Python Data Analysis Library (pandas, https://pandas.pydata.org/); the regressions and other statistical tests were run in Stata.

### 2.4 Data analysis

The effect of community composition and richness on the RGB profile was assessed, using the mean RGB value of each culture or its standardized value (minus sample average, divided by standard deviation). We examined the effect of several covariates on the maximum and minimum points of the temperature profiles, the (average) temperature after exposition to light, and the amplitude of the change before/after the light treatment—the linear regressions (ordinary least squares, OLS) are described below, and their results are detailed in the next section.

The aforementioned covariates include time trends and a measure of the selection and complementarity effects as defined in Loreau and Hector (2001). The time trends were meant to control for a possible warming over time of the flasks in the measurement room. To learn about the relationship between biovolume and RGB, we estimated the specification given in Equation 1:

(1) $${\text{RGB}}_{k}=\alpha_{0}+\alpha_{1}{\left(\text{Biovolume}\right)}_{k}+\sum_{f=1}^{F}\alpha_{f}{\left(\text{Functional Group}\right)}_{\mathrm{kf}}+\varepsilon_{k}$$

where (Biovolume)_*k*_ is the biovolume measured in flask *k*, (Functional Group)_*kf*_ is a dummy for the (initial) presence/absence of the functional group *f* in flask *k*, α_0_ is the intercept, and ε_*k*_ is the error term. The results are presented in Table 2 of the Results section.

To investigate the effect of each hypothesized causal mechanism of influence of biodiversity on temperature (namely, albedo, activity, and other unknown channels), we regressed each of our temperature variables on each of the suspected causes, as specified in Equation 2 in its most generic form and fullest specification:

(2) $$\begin{array}{cc} {\text{(Temp)}}_{k}=\alpha_{0} & +\sum_{f=1}^{F}\alpha_{f}\text{(Functional}\;{\text{Group)}}_{\mathrm{kf}}+\lambda_{1}{\text{Time}}_{k}\times {\left(\text{1st Batch}\right)}_{k}+\lambda_{2}{\text{Time}}_{k}\times {\left(\text{2nd Batch}\right)}_{k}\\ & +\gamma_{1}{\text{RGB}}_{k}+\gamma_{2}{\left({\text{RGB}}_{k}\right)}^{2}+\beta_{1}\text{(Complementarity}\;{\text{Effect)}}_{k}+\beta_{2}{\left(\text{Selection Effect}\right)}_{k}+\varepsilon_{k} \end{array}$$

where *Temp* stands for: Δ*T*,* T*_a_ (the temperature after), *T*_min_ or *T*_max_.

In Equation 2, (RGB)_*k*_ is the mean RGB value for flask *k*, (Temp)_*k*_ stands for either *T*_a_ (the average temperature of the content of flask *k* measured *after* exposure to the light source) or Δ*T* (the temperature *change* before/after exposure) or *T*_min_ or *T*_max_ (extreme values measured in the microcosm). (Functional Group)_*kf*_ is a dummy variable that receives a value of 1 if functional group *f* is present in flask *k* for all functional groups. Time_*k*_ is a linear time trend for the time at which the flask was analyzed (to account for heating of the room). (First Batch)_*k*_ and (Second Batch)_*k*_ are dummy variables that receive a value of 1 if the flask belongs to the first or second batch analyzed, respectively. Finally, ε_*k*_ is the error term, and α_0_ is the regression constant.

The results are discussed in the Results section.

## 3 RESULTS

### 3.1 Biodiversity significantly affects productivity

In this study, productivity is measured as the biovolume of the community after 13 days of growth with abundant light and nutrients. As shown on Table 1 and on Figure S5, the biovolume increases with the richness of the microcosm, but no single species has a significant effect on total biovolume (Table 1).

It should be noted (see Figure 2) that individual species behaviors are idiosyncratic: For instance, in *Ankistrodesmus* and *Selenastrum*, biovolume increases as the number of species increases, but *Cosmarium* did much better in monoculture than in coculture (in *Ankistrodesmus*, from a median biovolume of about 10^5^ μm^3^ in monoculture to about 3 × 10^5^ μm^3^ in the company of the seven other species, as opposed to *Cosmarium* starting in monoculture with a median biovolume of 3 × 10^5^μm^3^ and a fat upper tail, lower values at *n* = 2 and *n* = 4 and back to about 3 × 10^5^ μm^3^ with all 8 species). Interestingly, *Cosmarium* is the species with the highest unitary biovolume.

Our results are overall consistent with the widely observed positive saturating relationship between plant species richness and primary production (Cardinale et al., 2012; Tilman et al., 2014).

### 3.2 Biovolume significantly affects light absorption

Although it is likely that higher biovolumes would lead to greater visible‐spectrum absorbance, there is no reason a priori to assume that species‐specific volumes and pigment content are correlated. We tested this by estimating the regression described by Equation 1, where mean RGB is regressed against biovolume; the results are summarized in Table 2. Further illustration of this relationship is provided by Figure S6. The negative relationship between biovolume and RGB is tenuous yet visible on Figure S6a, reflecting the negative coefficient obtained in column (1) of Table 2. Figure S6b shows that this relationship persists even when the effect of individual species’ presence is controlled for; according to column (2) of Table 2, accounting species identity even strengthens and makes this negative effect become statistically significant at the 10% level. We note that while 0.1 is not frequently used as a significance level in ecology, (Yoccoz, 1991) notes that “there is nothing sacred about the value of 0.05” and that biological significance, rather than statistical significance (while necessary), should be emphasized.

Table 2 also points to the importance of some individual species: The presence of *Selenastrum* seems to increase reflectance—which is consistent with the fact that *Selenastrum* tended to thrive in any combination of species and therefore produced a lot of biovolume and opacity (apparently not at the expense of the other species)—and the presence of *Cosmarium* seems to decrease light absorption—which is consistent with our observation that *Cosmarium* did, at best, reproduce less than the other genera (and that competition with other genera was in general detrimental to it), thus making the microcosm not as opaque as it could have been.

Adding the initial richness of the microcosm to the initial specification explained more of the variance (Table S3 and Figure 3) than when solely considering the effect of biovolume; the importance of individual species is still supported (see Table S3, though *Cosmarium*'s influence is not significant anymore), but the negative effect of biodiversity (through higher productivity) on the RGB mean is no more. A positive slope is found with the linear specification (column (1) in Table S3 and green line on Figure 3), and a quadratic specification provides a better fit (column (2) in Table S3 and blue line on Figure 3), that is, RGB mean is high in monocultures, decreases in low‐diversity mixes, and increases in high‐diversity mixes.

Based on these results, the first channel (albedo) of influence of diversity on thermal properties seems valid (though somewhat complex) and appears to be mediated by the system's increased productivity (higher biovolume).

### 3.3 Local temperature is not directly influenced by biodiversity

We now turn to the effect of each hypothesized causal mechanism of influence of biodiversity on temperature and estimated the model described in Equation 2. The temperature variables we considered were *T*_a_ (the average temperature of the content of flask *k* measured *after* exposure to the light source), Δ*T* (the temperature *change* before/after exposure), *T*_min_, and *T*_max_ (extreme values measured in the microcosm).

We focus here on the results for Δ*T*, reported in Table 3. The regression tables for the other temperature variables and specifications are consigned in Tables S4–S7 (*T*_a_, Δ*T*,* T*_max_, and *T*_min_, respectively; no RGB), S8–S11 (idem; but linear in RGB), and S12–S14 (*T*_a_, *T*_max_, and *T*_min_, respectively; quadratic in RGB (full specification)) of the Supplementary Material. The distribution of the dependent variables *T*_a_, *T*_max_, and *T*_min_ is also available in Figure S4. Briefly, Tables S4, S8 and S12 show that under the specification used, only the time trends and the presence of *Selenastrum* have a robust and significant effect on the temperature of the microcosms after exposition to light (*T*_a_, *N* = 169). As regards the extreme values of the temperature distribution within the microcosm (*T*_min_ and *T*_max_, *N* = 169), while potentially of ecological significance, they do not seem to be affected in a robust manner by anything other than the time trend. These results are presented in Tables S6‐S7, S10‐S11, S13‐S14. While mean RGB and some genera (those with the largest contribution to biovolume) appear to have a significant effect under some specifications, these effects all disappear when the time trend is taken into account (compare columns (3) and (6) of Tables S10, S11, S13 and S14), or when the selection and complementarity effects are included, which makes the reality of these effects doubtful.

**Table 3:** Regression results: influence of microcosm composition and greenness on Δ*T*. Each column corresponds to a separate regression on Δ*T*: The independent variables are as follows: in column (1), a quadratic in RGB mean; in column (2), a quadratic in RGB mean and group dummies; in column (3), a quadratic in RGB mean, group dummies, and a time trend; in column (4), selection and complementarity effects only; in column (5), a quadratic in RGB mean, group dummies, selection, and complementarity effects; and in column (6), a quadratic in RGB mean, group dummies, a time trend, selection, and complementarity effects

Dependent Variable: Temperature difference (°C)						
	(1)	(2)	(3)	(4)	(5)	(6)
(RGB Mean)	0.068 (0.066)	0.202*** (0.071)	0.200*** (0.071)		0.180** (0.089)	0.184** (0.089)
(RGB Mean)^2^	−0.000 (0.000)	−0.001*** (0.000)	−0.001*** (0.000)		−0.001** (0.000)	−0.001** (0.000)
*Ankistrodesmus*		0.007 (0.025)	0.008 (0.025)		−0.002 (0.031)	−0.001 (0.031)
*Cosmarium*		−0.027 (0.025)	−0.024 (0.026)		−0.023 (0.032)	−0.016 (0.034)
*Selenastrum*		−0.008 (0.028)	−0.003 (0.031)		0.003 (0.031)	0.011 (0.035)
*Staurastrum*		−0.006 (0.027)	−0.001 (0.028)		−0.007 (0.030)	−0.000 (0.031)
Isomorphic group		−0.128*** (0.043)	−0.121*** (0.045)		−0.097 (0.073)	−0.083 (0.072)
Time trend second batch			−0.000 (0.000)			−0.000 (0.000)
Selection effect				0.000 (0.000)	−0.000 (0.000)	−0.000 (0.000)
Complementarity effect				0.000 (0.000)	−0.000 (0.000)	−0.000 (0.000)
Constant	−4.868 (4.620)	−14.029*** (4.968)	−13.845*** (4.952)	0.015 (0.022)	−12.566** (6.168)	−12.849** (6.213)
*R* ^2^	0.019	0.121	0.123	0.006	0.078	0.081
*N*	109	109	109	84	84	84

Robust standard errors in parentheses.

* *p* < 0.1

** *p* < 0.05

*** *p* < 0.01.

If we restrict our analysis to the data of the second batch (*N* = 109), we can compute the temperature difference Δ*T* (before/after exposition to the source of light), which is more relevant to a variable, and proceed to similar regressions (specification following Equation 2 with Δ*T* as the dependent variable), whose results are reported in Table 3.

Temperature difference is not, unlike the other temperature variables, affected by the warming of the room (the time trend). Rather, as can be seen in Table 3, the reflectance of the suspension and the presence of the isomorphic functional group (IG) are the main drivers of the change in temperature due to exposition to light. The effect of the functional group disappears when selection and complementarity effects are included, but this may be caused by the loss of 20 samples (the monocultures and the controls, for which these variables cannot be computed), thus decreasing statistical power and possibly blurring the picture as a result (lower *R* ^2^). We note that the exclusion of 20 samples is likely to have reduced statistical power, so we are cautious in our interpretation of the results. The presence of elements of the IG functional group in the microcosm decreases the temperature change, and this effect seems robust to addition/deletion of controls, see also Table S5. The RGB mean (“albedo”) is also an important driver of the magnitude of the temperature change. However, none of the biodiversity effects is significant, in any regression specification we tried.

**Figure 4:**
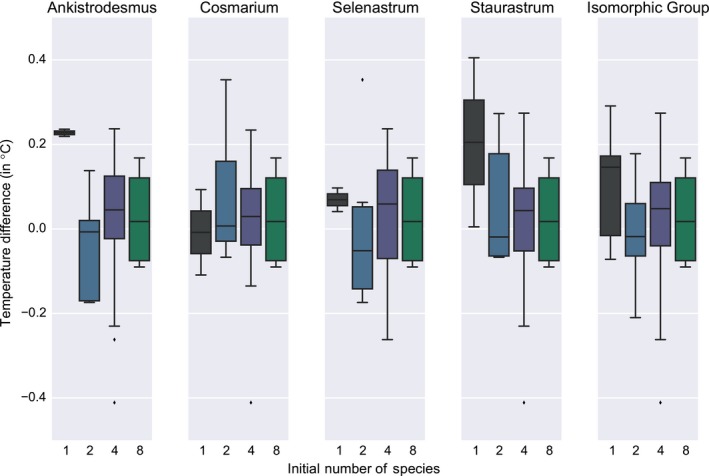
Flask change in temperature, per species. Each column presents the change in temperature (before/after exposition to light) of the flasks containing a particular species, be it as a monoculture or in an assemblage of 2, 4, and 8 species. The horizontal line corresponds to the median, the box shows the quartiles, the whiskers describe the rest of the distribution, and the points beyond the whiskers are outliers

This absence of a distinct, one‐sided, biodiversity effect is also visible in Figure 4: No clear pattern pertaining to the number of species emerges but for the fact that monoculture extremes (encountered for instance with *Ankistrodesmus*,* Selenastrum*, and *Staurastrum*) are tempered by the addition of other species. This seems not to be a dilution effect, judging by the differences between *n* = 1 and *n* = 2 for those species. Therefore, if anything, biodiversity, in our microcosm experiment, dampens the thermal properties of the community.

**Figure 5:**
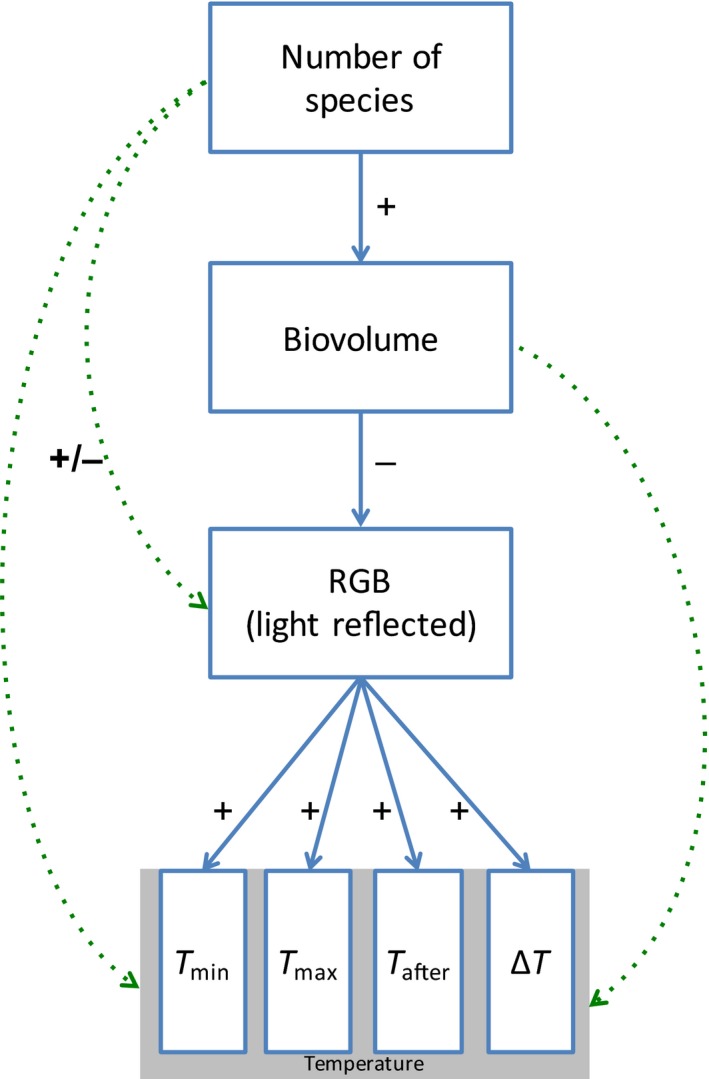
Summary schematic of collective findings and conclusions. Solid lines represent the statistically significant and unambiguous results, with a plus or minus sign representing the type of the relationship. Dotted lines represent the nonsignificant relationships. In our microcosms, increased species richness led to an increased biovolume, which in turn led to higher mean RGB (reflectance) values, and higher mean RGB values were significantly associated with higher thermal outcomes: higher Δ*T* (the temperature difference before/after exposition to light), higher *T*_a_ (the temperature difference after exposition to light), and higher *T*_min_ and *T*_max_ (local extrema)

Figure 5 summarizes our findings. An increase in species richness increases biovolume (with a constant number of cells at time *t* = 0); an increase in biovolume decreases the mean RGB value; and a decrease in RGB is associated with a decrease in temperature (or possibly an increase in temperature change). However, no empirical evidence supports any effect of biovolume on temperature (change), nor of biodiversity on temperature (change) directly.

## 4 DISCUSSION

Algal species richness in this microcosm study exhibited the positive relationship with primary production observed in many BEF experiments, but showed no direct relationship with the local thermal environmental properties, assessed in this case as the change in temperature, measured by thermography, that occurred after a 60‐s exposure to light. Primary production, or algal community biovolume, also did not show a positive relationship with local thermal environmental properties. Instead, the likely causal chain of the influence of diversity over the local thermal environment is through its impact on biovolume and RGB reflectance (color). Figure 5 summarizes these relationships.

Our study focuses on these issues and illustrates both the approach and complications one may encounter in attempting to identify biodiversity effects that may be subtle or otherwise difficult to detect. We were able to generate a diversity effect on production, as many BEF experiments have found in the past; this change in production had an effect on visible‐spectrum absorbance (or, its inverse, reflection, which we measured through RGB imagery). The diversity‐induced change in absorbance did impact local temperature, but the effects were weak (low *R* ^2^s) and ultimately did not provide a statistically significant link between biodiversity and the local thermal environment (Figure 5). The fact that we were unable to find any effect of biodiversity and biovolume on temperature (despite the other relationships found) could indicate that there is indeed no effect or that our sample size was too small and our experimental protocols too imprecise (in particular, the fact that we were unable to discriminate between genera of the “IG” functional group). In addition, it should be kept in mind that, as any typical BEF experiment, this protocol does not enable to distinguish between “noise” variation (measurement error, etc.) and variation caused by community composition, the latter of which is at play in communities made up of 1–4 species, but not 8 (all species).

Given the challenges of measuring potentially subtle effects in algal communities, if one considers that small changes in temperature affect numerous microbial processes in phytoplankton and their associated microbial communities, our findings potentially touch upon important possibilities for the impacts of changing biodiversity on ecosystem functions and properties. It has also been recently noted that microcosm experiments manipulating biodiversity tended to underestimate outcomes occurring in the wild (in terms of community production and stability; Duffy, Godwin, & Cardinale, 2017). Given the vast surface area of freshwater and marine systems and the clarity of the mechanism we identified, even though the direct linkage between diversity and temperature was difficult to detect, the implications are clear. Our results should encourage an alteration in the way albedo is modeled and go beyond the uniform and time‐invariant value attributed to bodies of water and, more generally, to biomes. Such diversity effects could translate to important temperature‐mediated biogeochemical consequences at large scales in our world where changes in climate and biodiversity are co‐occurring.

## CONFLICT OF INTEREST

The authors declare no competing interests.

## Data Availability

Experimental data necessary to reproduce the analyses are made available on Dryad: https://doi.org/10.5061/dryad.59p3vv5 (Missirian et al. 2019).
