# Supplementary material for: Biodiversity and thermal ecological function: The influence of freshwater algal diversity on local thermal environments
Source: Ecol Evol. 2019 May 22;9(12):6949–58. doi: 10.1002/ece3.5262 (PMC6662266; doi:10.1002/ece3.5262)
Supplement: Supplementary file 1 [file ECE3-9-6949-s001.pdf]

Supporting Information

Additional experimental details

Timing of the experiment The first batch of microcosms was assembled on November 26th, 2014, and the last series of microcosms were imaged on December 15th, 2014, allowing for a 13 day incubation period for all the microcosms.

Sampling schedule and processing The first batch of microcosms was prepared on November 26th, 2014, and sacrificed 13 days later (i.e. December 9th): thermal and visible imagery was conducted, and samples were subsequently disposed of. The second and third batches were prepared in the same fashion together on December 2nd, 2014, and imaged together on December 15th, 13 days after assembly as well. Thirteen days was the duration required to ascertain reasonable (observable) growth was taking place; it is consistent with the duration specified on growth medium for "most algae [to] show substantial growth". Before assembly, the pure algal strands were stored at room temperature under a 24 W T5 fluorescent lamp. After assembly, the microcosms were stored at room temperature and were shuffled daily to homogenize exposition to light.

S1 Table

Table S1. Community combinatorics.

	C_n^k	replicates	nb. microcosms
0 species (control)	1		5
1 species	8	8 triplicates	24
2 species	28	12 duplicates, 16 singles	70
4 species	70	24 duplicates, 46 singles	54
8 species	1	1 sextuplicate	6
Total			169

Species information

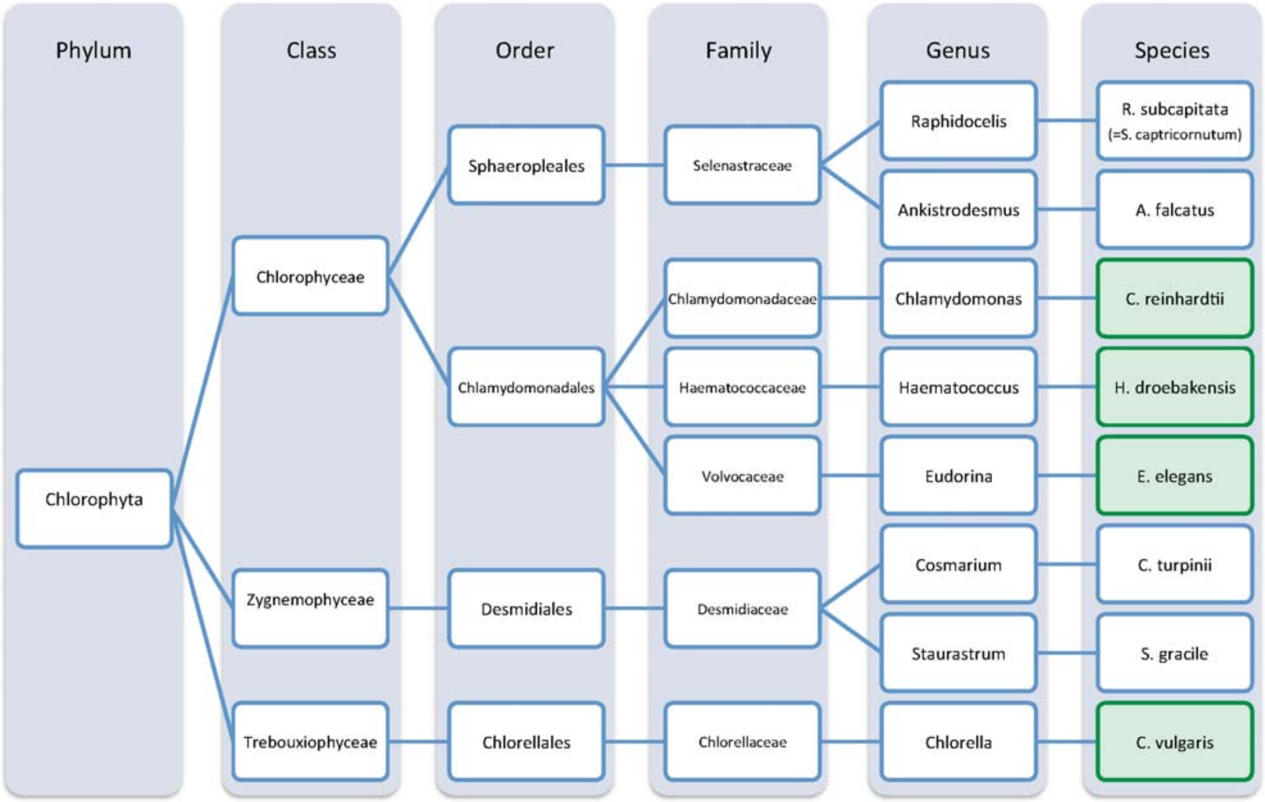

Figure S1. Phylogenetic information.

Phylogenetic relationships of the eight species used. Shadowed in green: the four isomorphic species.

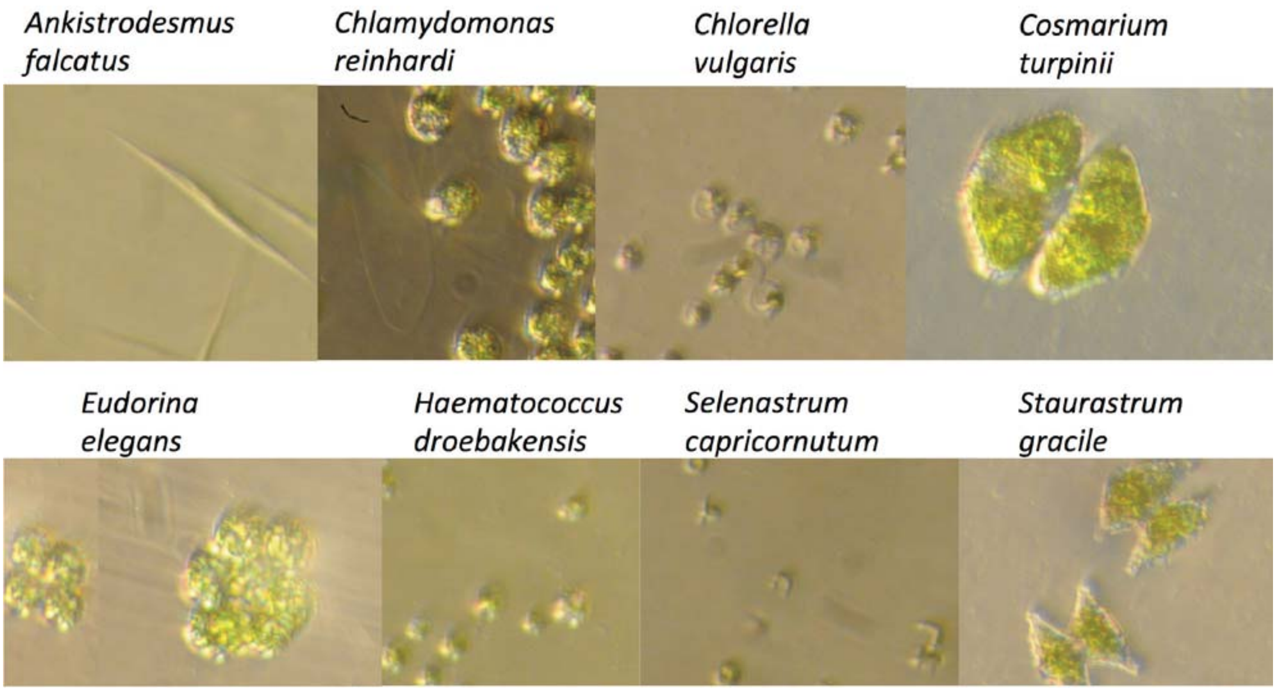

Figure S2. Algal morphology.

Plate used for species identification.

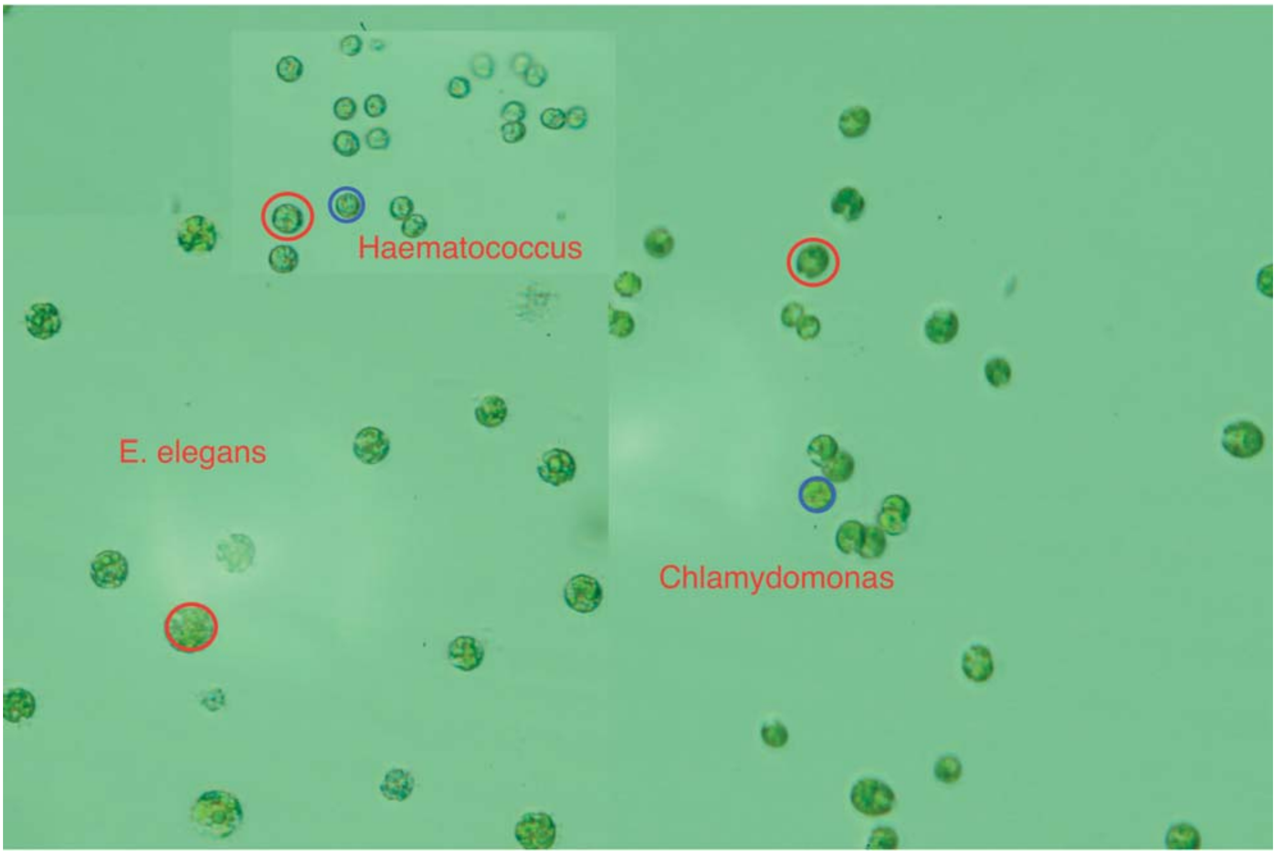

Figure S3. Algal morphology, experimental conditions.

Mosaic of photographs of slides under inverted microscope at magnification 40x, used for enumeration. Annotated to highlight morphological closeness between *Chlamydomonas*, *E. elegans*, *Haematococcus*.

Empirical biovolumes

Table S2. Biovolumes

Species	Biovolume (μm^3)	replicates	Std. err.
Ankistrodesmus falcatus	116	75	9.9
Chlamydomonas reinhardtii	313	75	21.7
Chlorella vulgaris	95	75	8.1
Cosmarium turpinii	33 226	20	2 355.8
Eudorina elegans	456	75	46.2
Haematococcus droebakensis	154	75	10.2
Selenastrum capricornutum	45	75	2.9
Staurastrum gracile	2 882	20	196.5

Temperature distributions in the sample

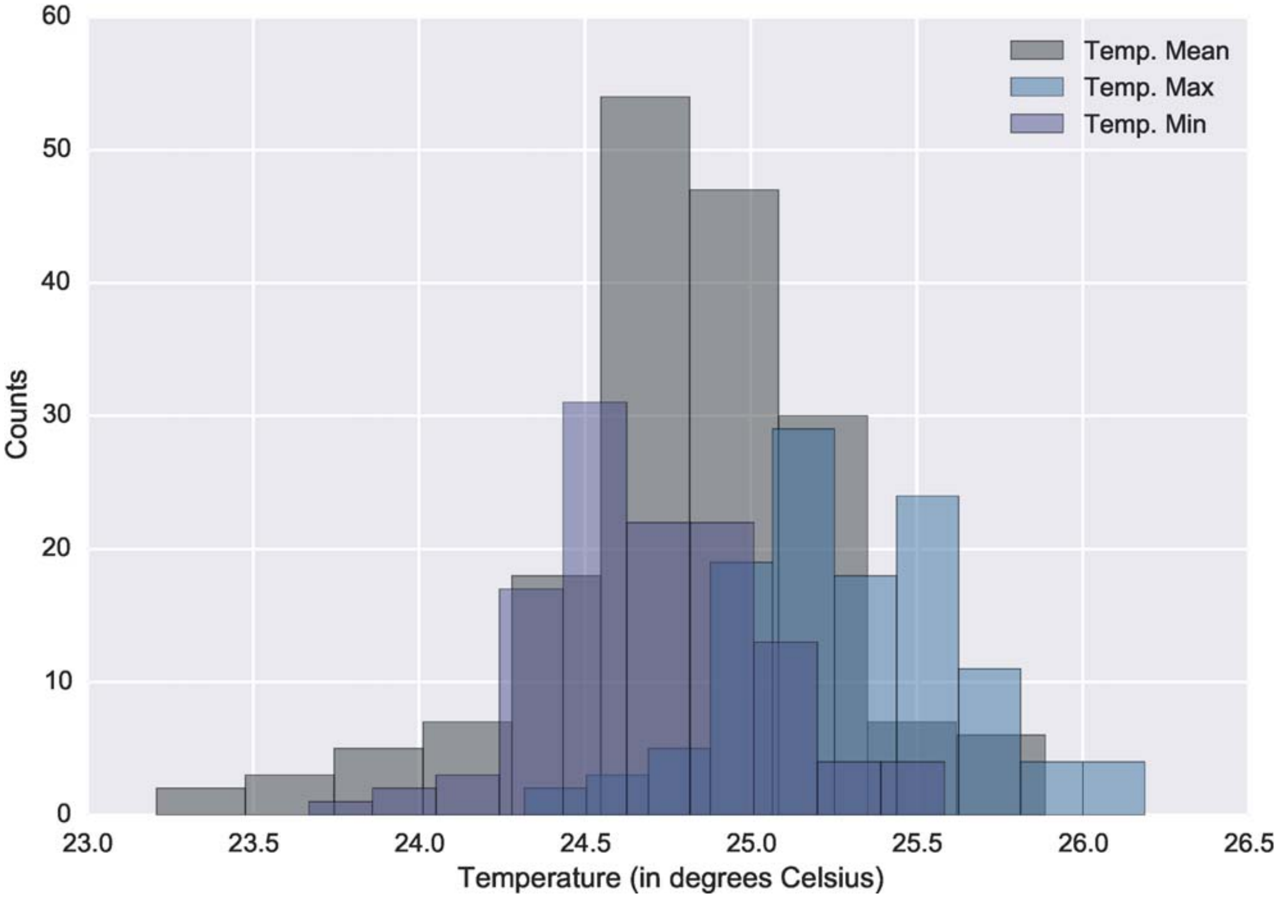

Figure S4. Distribution of temperature (min, max, average) in the sample.

Species richness is positively correlated to biovolume

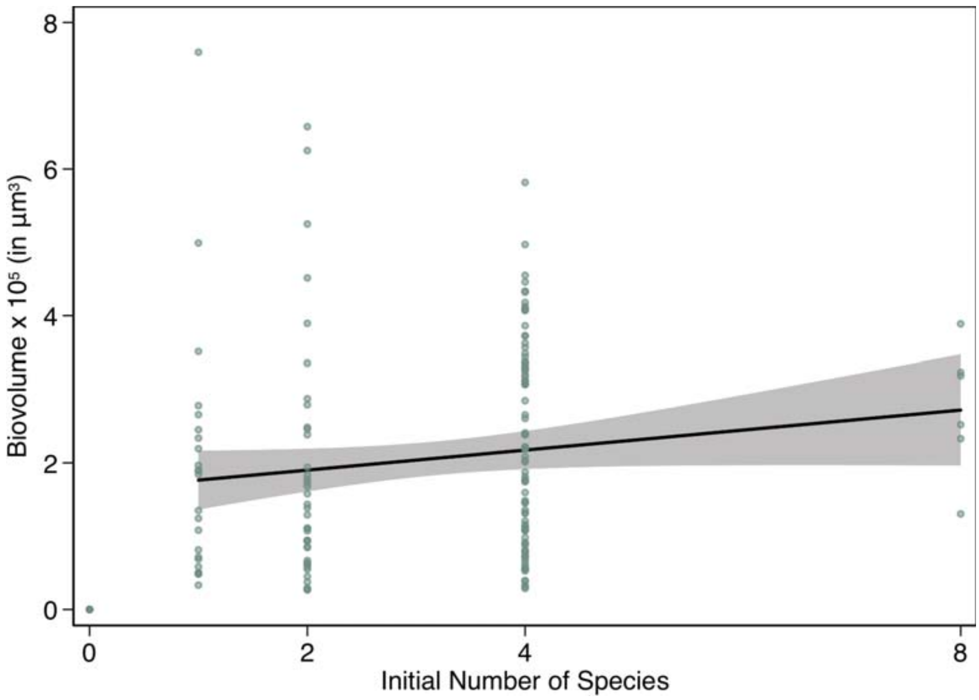

Figure S5. Microcosm biovolume and initial species richness
Solid line represents the linear best fit of the measured biovolume as a function of the number of initial species. Shaded area represents 95% confidence intervals.

Biovolume is negatively correlated to RGB

In both regressions presented in Table 2, biovolume is negatively associated with RGB values (albeit insignificantly so in Column (1)). Column (2) shows that this relationship persists, and is strengthened (i.e. becomes significant at the 10% level), when the effect of individual species' presence is controlled for. Note that Column (2) also points to the importance of some individual species: the presence of *Selenastrum* seems to increase "greenness" – which is consistent with the fact that *Selenastrum* tended to thrive in any combination of species, and therefore produced a lot of biovolume and opacity (apparently not at the expense of the other species) – and the presence of *Cosmarium* seems to decrease light absorption – which is consistent with our observation that *Cosmarium* did, at best, reproduce less than the other genera (and that competition with other genera was in general detrimental to it), thus making the microcosm not as opaque as it could have been.

Fig. S6a shows the RGB values versus the biovolume (analogous to Column (1) of Table 2), and hints at a negative but weak relationship between biovolume and RGB; on Fig. S6b are plotted the residuals from the regression of RGB on the functional group dummies, against the residuals from biovolume on the functional group dummies – i.e. a partial regression plot – which shows that the negative slope persists even when the effect of individual species' presence is subtracted from both biovolume and RGB (mirroring the findings of Column (2) in Table 2).

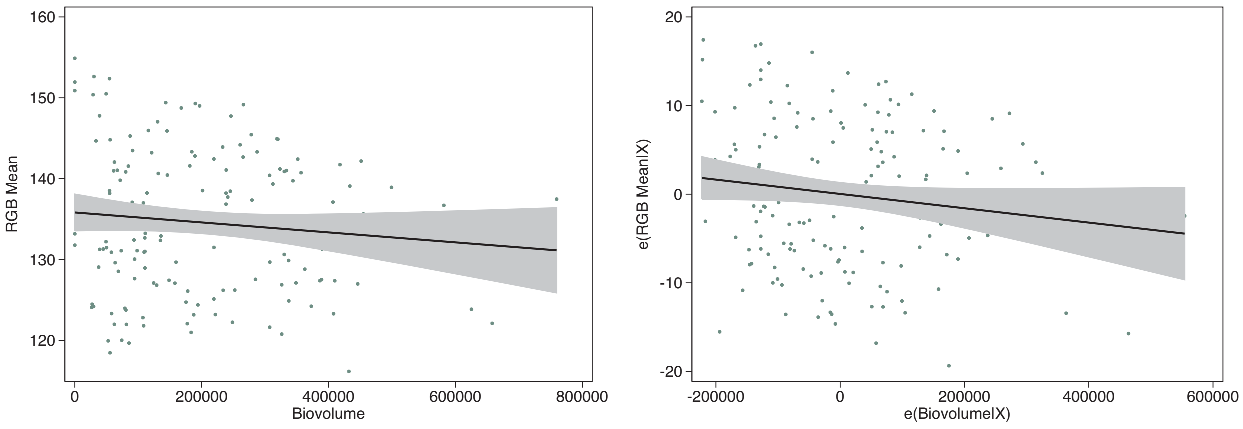

(a) Scatter Plot **(b)** Partial Regression Plot

Figure S6. Influence of biovolume on RGB Mean

Scatter plot and the partial regression plot showing the negative linear relationship between the mean RGB and biovolume. Solid line shows the linear best fit. Shaded area shows the 95% confidence intervals.

There is a quadratic relationship between species richness and RGB

Table S3. Regression results: Microcosm composition and RGB mean value.

Notes: $*p < 0.1, **p < 0.05, ***p < 0.01$

Dependent Variable: RGB Mean

	(1)	(2)
$N_{Species}$	1.487**	-1.315
	(0.750)	(1.431)
$N^2_{Species}$		0.330**
		(0.162)
Ankistrodesmus	-3.682**	-3.170**
	(1.521)	(1.502)
Cosmarium	0.297	0.846
	(1.511)	(1.497)
Selenastrum	-4.827***	-4.402***
	(1.358)	(1.366)
Staurastrum	-1.310	-0.862
	(1.644)	(1.641)
Isomorphic Group	-5.310**	-3.069
	(2.474)	(2.469)
Constant	138.099***	140.139***
	(2.097)	(2.312)
R^2	0.100	0.123
N	169	169

Other regressions

Table S4. Regression results: Microcosm composition and T_a.

Notes: $*p < 0.1, **p < 0.05, ***p < 0.01$. Robust standard errors in parentheses.

Dependent Variable: Temperature After (°C)

	(1)	(2)	(3)	(4)	(5)
Ankistrodesmus	-0.085	-0.104*		-0.082	-0.093
	(0.070)	(0.053)		(0.087)	(0.071)
Cosmarium	-0.004	-0.047		0.023	-0.045
	(0.071)	(0.059)		(0.082)	(0.072)
Selenastrum	0.102	0.120**		0.115	0.137*
	(0.070)	(0.059)		(0.079)	(0.072)
Staurastrum	0.088	0.079		0.115	0.090
	(0.069)	(0.062)		(0.085)	(0.077)
Isomorphic Group	-0.020	-0.001		0.000	-0.009
	(0.060)	(0.070)		(0.114)	(0.120)
Time Trend First Batch		-0.001***			-0.001**
		(0.000)			(0.000)
Time Trend Second Batch		0.000***			0.001***
		(0.000)			(0.000)
Selection Effect			-0.000	-0.000	0.000
			(0.000)	(0.000)	(0.000)
Complementarity Effect			-0.000	-0.000	0.000
			(0.000)	(0.000)	(0.000)
Constant	24.779***	24.726***	24.846***	24.771***	24.629***
	(0.058)	(0.066)	(0.074)	(0.153)	(0.156)
R^2	0.030	0.396	0.009	0.048	0.429
N	169	169	129	129	129

Table S5. Regression results: Microcosm composition and ΔT.

Notes: $*p < 0.1, **p < 0.05, ***p < 0.01$. Robust standard errors in parentheses.

Dependent Variable: Temperature Difference (°C)

	(1)	(2)	(3)	(4)	(5)
Ankistrodesmus	-0.006	-0.005		-0.011	-0.011
	(0.027)	(0.027)		(0.031)	(0.031)
Cosmarium	-0.015	-0.012		-0.002	0.000
	(0.025)	(0.027)		(0.032)	(0.034)
Selenastrum	-0.010	-0.007		0.002	0.003
	(0.027)	(0.028)		(0.029)	(0.032)
Staurastrum	0.002	0.006		0.001	0.003
	(0.027)	(0.030)		(0.029)	(0.032)
Isomorphic Group	-0.081*	-0.076*		-0.052	-0.048
	(0.043)	(0.043)		(0.080)	(0.080)
Time Trend Second Batch		-0.000			-0.000
		(0.000)			(0.000)
Selection Effect			0.000	0.000	0.000
			(0.000)	(0.000)	(0.000)
Complementarity Effect			0.000	0.000	0.000
			(0.000)	(0.000)	(0.000)
Constant	0.116***	0.124**	0.015	0.066	0.068
	(0.043)	(0.049)	(0.022)	(0.091)	(0.094)
R^2	0.057	0.058	0.006	0.019	0.020
N	109	109	84	84	84

Table S6. Regression results: Microcosm composition and T_{max}.

Notes: $*p < 0.1, **p < 0.05, ***p < 0.01$. Robust standard errors in parentheses.

Dependent Variable: Temperature Maximum (°C)

	(1)	(2)	(3)	(4)	(5)
Ankistrodesmus	-0.094	-0.114**		-0.092	-0.105
	(0.072)	(0.053)		(0.089)	(0.072)
Cosmarium	-0.001	-0.053		0.029	-0.055
	(0.073)	(0.059)		(0.084)	(0.071)
Selenastrum	0.093	0.106*		0.100	0.113
	(0.072)	(0.058)		(0.081)	(0.072)
Staurastrum	0.080	0.063		0.105	0.067
	(0.071)	(0.062)		(0.088)	(0.078)
Isomorphic Group	-0.020	-0.007		0.016	-0.018
	(0.062)	(0.067)		(0.123)	(0.131)
Time Trend First Batch		-0.001***			-0.001**
		(0.000)			(0.000)
Time Trend Second Batch		0.001***			0.001***
		(0.000)			(0.000)
Selection Effect			-0.000	-0.000	0.000
			(0.000)	(0.000)	(0.000)
Complementarity Effect			-0.000	-0.000	0.000
			(0.000)	(0.000)	(0.000)
Constant	25.098***	25.033***	25.162***	25.089***	24.931***
	(0.060)	(0.064)	(0.076)	(0.162)	(0.163)
R^2	0.026	0.427	0.010	0.044	0.448
N	169	169	129	129	129

Table S7. Regression results: Microcosm composition and T_{min}.

Notes: $*p < 0.1, **p < 0.05, ***p < 0.01$. Robust standard errors in parentheses.

Dependent Variable: Temperature Minimum (°C)

	(1)	(2)	(3)	(4)	(5)
Ankistrodesmus	-0.069	-0.085		-0.065	-0.075
	(0.066)	(0.052)		(0.082)	(0.069)
Cosmarium	-0.024	-0.059		-0.001	-0.065
	(0.068)	(0.058)		(0.078)	(0.070)
Selenastrum	0.086	0.105*		0.096	0.114
	(0.066)	(0.058)		(0.074)	(0.072)
Staurastrum	0.079	0.075		0.104	0.079
	(0.066)	(0.061)		(0.081)	(0.076)
Isomorphic Group	-0.026	-0.005		-0.013	-0.026
	(0.057)	(0.068)		(0.105)	(0.114)
Time Trend First Batch		-0.001***			-0.001**
		(0.000)			(0.000)
Time Trend Second Batch		0.000***			0.001***
		(0.000)			(0.000)
Selection Effect			-0.000	-0.000	0.000
			(0.000)	(0.000)	(0.000)
Complementarity Effect			-0.000	-0.000	0.000
			(0.000)	(0.000)	(0.000)
Constant	24.546***	24.505***	24.585***	24.535***	24.406***
	(0.055)	(0.064)	(0.071)	(0.141)	(0.146)
R^2	0.025	0.346	0.008	0.040	0.382
N	169	169	129	129	129

Table S8. Regression results: mean RGB and T_a (linear).

Notes: $*p < 0.1, **p < 0.05, ***p < 0.01$. Robust standard errors in parentheses.

Dependent Variable: Temperature After (°C)

	(1)	(2)	(3)	(4)	(5)	(6)
(RGB Mean)	0.020***	0.022***	-0.001		0.027***	-0.001
	(0.004)	(0.004)	(0.005)		(0.005)	(0.006)
Ankistrodesmus		-0.038	-0.108*		-0.066	-0.093
		(0.063)	(0.056)		(0.079)	(0.072)
Cosmarium		-0.049	-0.048		-0.074	-0.044
		(0.065)	(0.059)		(0.073)	(0.072)
Selenastrum		0.176***	0.115*		0.173**	0.135*
		(0.066)	(0.064)		(0.075)	(0.077)
Staurastrum		0.081	0.077		0.083	0.089
		(0.061)	(0.063)		(0.073)	(0.078)
Isomorphic Group		0.039	-0.006		-0.051	-0.009
		(0.073)	(0.070)		(0.173)	(0.119)
Time Trend First Batch			-0.001***			-0.001**
			(0.000)			(0.000)
Time Trend Second Batch			0.000***			0.001**
			(0.000)			(0.000)
Selection Effect				-0.000	-0.000	0.000
				(0.000)	(0.000)	(0.000)
Complementarity Effect				-0.000	-0.000	0.000
				(0.000)	(0.000)	(0.000)
Constant	22.084***	21.689***	24.918***	24.846***	21.166***	24.727***
	(0.489)	(0.550)	(0.635)	(0.074)	(0.659)	(0.787)
R^2	0.157	0.207	0.396	0.009	0.269	0.429
N	169	169	169	129	129	129

Table S9. Regression results: mean RGB and ΔT (linear).

Notes: $*p < 0.1, **p < 0.05, ***p < 0.01$. Robust standard errors in parentheses.

Dependent Variable: Temperature Difference (°C)

	(1)	(2)	(3)	(4)	(5)	(6)
(RGB Mean)	0.002	0.002	0.002		0.003	0.003
	(0.002)	(0.002)	(0.002)		(0.003)	(0.003)
Ankistrodesmus		-0.004	-0.002		-0.012	-0.011
		(0.026)	(0.026)		(0.031)	(0.031)
Cosmarium		-0.019	-0.014		-0.013	-0.007
		(0.026)	(0.027)		(0.033)	(0.034)
Selenastrum		-0.003	0.005		0.010	0.016
		(0.028)	(0.031)		(0.030)	(0.035)
Staurastrum		-0.001	0.006		-0.006	-0.000
		(0.028)	(0.030)		(0.030)	(0.032)
Isomorphic Group		-0.077*	-0.067		-0.058	-0.046
		(0.044)	(0.045)		(0.078)	(0.078)
Time Trend Second Batch			-0.000			-0.000
			(0.000)			(0.000)
Selection Effect				0.000	-0.000	0.000
				(0.000)	(0.000)	(0.000)
Complementarity Effect				0.000	0.000	0.000
				(0.000)	(0.000)	(0.000)
Constant	-0.242	-0.106	-0.170	0.015	-0.343	-0.371
	(0.274)	(0.304)	(0.324)	(0.022)	(0.355)	(0.374)
R^2	0.010	0.062	0.066	0.006	0.036	0.038
N	109	109	109	84	84	84

Table S10. Regression results: mean RGB and T_{max} (linear).

Notes: $*p < 0.1, **p < 0.05, ***p < 0.01$. Robust standard errors in parentheses.

Dependent Variable: Temperature Maximum (°C)

	(1)	(2)	(3)	(4)	(5)	(6)
(RGB Mean)	0.022***	0.024***	-0.001		0.029***	-0.001
	(0.004)	(0.004)	(0.005)		(0.005)	(0.006)
Ankistrodesmus		-0.043	-0.117**		-0.076	-0.105
		(0.063)	(0.055)		(0.080)	(0.072)
Cosmarium		-0.049	-0.053		-0.075	-0.054
		(0.066)	(0.059)		(0.075)	(0.071)
Selenastrum		0.173**	0.102		0.162**	0.111
		(0.067)	(0.064)		(0.076)	(0.077)
Staurastrum		0.073	0.062		0.070	0.066
		(0.063)	(0.063)		(0.075)	(0.079)
Isomorphic Group		0.044	-0.011		-0.039	-0.018
		(0.074)	(0.067)		(0.178)	(0.130)
Time Trend First Batch			-0.001***			-0.001**
			(0.000)			(0.000)
Time Trend Second Batch			0.001***			0.001***
			(0.000)			(0.000)
Selection Effect				-0.000	-0.000	0.000
				(0.000)	(0.000)	(0.000)
Complementarity Effect				-0.000	-0.000	0.000
				(0.000)	(0.000)	(0.000)
Constant	22.123***	21.737***	25.179***	25.162***	21.254***	25.030***
	(0.496)	(0.555)	(0.634)	(0.076)	(0.670)	(0.788)
R^2	0.181	0.227	0.427	0.010	0.281	0.448
N	169	169	169	129	129	129

Table S11. Regression results: mean RGB and T_{min} (linear).

Notes: $*p < 0.1, **p < 0.05, ***p < 0.01$. Robust standard errors in parentheses.

Dependent Variable: Temperature Minimum (°C)

	(1)	(2)	(3)	(4)	(5)	(6)
(RGB Mean)	0.018***	0.020***	-0.001		0.025***	0.000
	(0.004)	(0.004)	(0.005)		(0.005)	(0.006)
Ankistrodesmus		-0.028	-0.087		-0.051	-0.075
		(0.060)	(0.055)		(0.075)	(0.070)
Cosmarium		-0.064	-0.059		-0.090	-0.065
		(0.062)	(0.058)		(0.070)	(0.071)
Selenastrum		0.151**	0.103		0.149**	0.114
		(0.063)	(0.064)		(0.071)	(0.076)
Staurastrum		0.073	0.074		0.075	0.079
		(0.059)	(0.062)		(0.070)	(0.077)
Isomorphic Group		0.026	-0.007		-0.059	-0.026
		(0.071)	(0.069)		(0.160)	(0.115)
Time Trend First Batch			-0.001***			-0.001**
			(0.000)			(0.000)
Time Trend Second Batch			0.000**			0.001**
			(0.000)			(0.000)
Selection Effect				-0.000	-0.000	0.000
				(0.000)	(0.000)	(0.000)
Complementarity Effect				-0.000	0.000	0.000
				(0.000)	(0.000)	(0.000)
Constant	22.139***	21.793***	24.603***	24.585***	21.273***	24.403***
	(0.476)	(0.530)	(0.646)	(0.071)	(0.622)	(0.804)
R^2	0.138	0.183	0.346	0.008	0.244	0.382
N	169	169	169	129	129	129

Table S12. Regression results: mean RGB and T_a (quadratic).

Notes: $*p < 0.1, **p < 0.05, ***p < 0.01$. Robust standard errors in parentheses.

Dependent Variable: Temperature After (°C)

	(1)	(2)	(3)	(4)	(5)	(6)
(RGB Mean)	0.338***	0.325***	0.098		0.398***	0.114
	(0.094)	(0.101)	(0.098)		(0.137)	(0.124)
$(\text{RGB Mean})^2$	-0.001***	-0.001***	-0.000		-0.001***	-0.000
	(0.000)	(0.000)	(0.000)		(0.001)	(0.000)
Ankistrodesmus		-0.019	-0.098*		-0.047	-0.086
		(0.063)	(0.056)		(0.077)	(0.072)
Cosmarium		-0.042	-0.047		-0.053	-0.039
		(0.063)	(0.059)		(0.073)	(0.071)
Selenastrum		0.148**	0.107*		0.136*	0.126
		(0.065)	(0.065)		(0.074)	(0.077)
Staurastrum		0.105*	0.083		0.109	0.098
		(0.060)	(0.063)		(0.073)	(0.080)
Isomorphic Group		-0.020	-0.025		-0.096	-0.023
		(0.070)	(0.072)		(0.153)	(0.120)
Time Trend First Batch			-0.001***			-0.001**
			(0.000)			(0.000)
Time Trend Second Batch			0.000***			0.001**
			(0.000)			(0.000)
Selection Effect				-0.000	-0.000	0.000
				(0.000)	(0.000)	(0.000)
Complementarity Effect				-0.000	-0.000	0.000
				(0.000)	(0.000)	(0.000)
Constant	0.679	1.370	18.163***	24.846***	-3.518	16.972**
	(6.390)	(6.834)	(6.666)	(0.074)	(9.190)	(8.320)
R^2	0.204	0.246	0.400	0.009	0.312	0.432
N	169	169	169	129	129	129

Table S13. Regression results: mean RGB and T_{max} (quadratic).

Notes: $*p < 0.1, **p < 0.05, ***p < 0.01$. Robust standard errors in parentheses.

Dependent Variable: Temperature Maximum (°C)

	(1)	(2)	(3)	(4)	(5)	(6)
(RGB Mean)	0.342***	0.327***	0.087		0.407***	0.105
	(0.095)	(0.102)	(0.096)		(0.139)	(0.123)
$(\text{RGB Mean})^2$	-0.001***	-0.001***	-0.000		-0.001***	-0.000
	(0.000)	(0.000)	(0.000)		(0.001)	(0.000)
Ankistrodesmus		-0.024	-0.109*		-0.057	-0.098
		(0.063)	(0.056)		(0.079)	(0.072)
Cosmarium		-0.042	-0.052		-0.053	-0.049
		(0.064)	(0.059)		(0.075)	(0.071)
Selenastrum		0.145**	0.095		0.124*	0.103
		(0.066)	(0.064)		(0.074)	(0.077)
Staurastrum		0.096	0.067		0.096	0.074
		(0.061)	(0.063)		(0.075)	(0.080)
Isomorphic Group		-0.016	-0.028		-0.084	-0.032
		(0.072)	(0.069)		(0.160)	(0.133)
Time Trend First Batch			-0.001***			-0.001**
			(0.000)			(0.000)
Time Trend Second Batch			0.001***			0.001***
			(0.000)			(0.000)
Selection Effect				-0.000	-0.000	0.000
				(0.000)	(0.000)	(0.000)
Complementarity Effect				-0.000	-0.000	0.000
				(0.000)	(0.000)	(0.000)
Constant	0.566	1.444	19.194***	25.162***	-3.894	17.871**
	(6.437)	(6.930)	(6.513)	(0.076)	(9.294)	(8.286)
R^2	0.227	0.264	0.430	0.010	0.324	0.451
N	169	169	169	129	129	129

Table S14. Regression results: mean RGB and T_{min} (quadratic).

Notes: $*p < 0.1, **p < 0.05, ***p < 0.01$. Robust standard errors in parentheses.

Dependent Variable: Temperature Minimum (°C)

	(1)	(2)	(3)	(4)	(5)	(6)
(RGB Mean)	0.295***	0.287***	0.085		0.331**	0.078
	(0.090)	(0.097)	(0.097)		(0.135)	(0.124)
$(\text{RGB Mean})^2$	-0.001***	-0.001***	-0.000		-0.001**	-0.000
	(0.000)	(0.000)	(0.000)		(0.001)	(0.000)
Ankistrodesmus		-0.011	-0.079		-0.036	-0.070
		(0.060)	(0.055)		(0.074)	(0.070)
Cosmarium		-0.057	-0.058		-0.072	-0.061
		(0.061)	(0.058)		(0.071)	(0.070)
Selenastrum		0.127**	0.096		0.119*	0.109
		(0.063)	(0.064)		(0.070)	(0.076)
Staurastrum		0.094	0.079		0.096	0.085
		(0.059)	(0.062)		(0.071)	(0.078)
Isomorphic Group		-0.026	-0.024		-0.096	-0.035
		(0.068)	(0.072)		(0.145)	(0.116)
Time Trend First Batch			-0.001***			-0.001*
			(0.000)			(0.000)
Time Trend Second Batch			0.000**			0.000**
			(0.000)			(0.000)
Selection Effect				-0.000	-0.000	0.000
				(0.000)	(0.000)	(0.000)
Complementarity Effect				-0.000	-0.000	0.000
				(0.000)	(0.000)	(0.000)
Constant	3.506	3.914	18.766***	24.585***	0.909	19.177**
	(6.089)	(6.546)	(6.591)	(0.071)	(9.024)	(8.332)
R^2	0.178	0.216	0.349	0.008	0.277	0.384
N	169	169	169	129	129	129
